# Key players of immunosuppression in epithelial malignancies: Tumor‐infiltrating myeloid cells and γδ T cells

**DOI:** 10.1002/cnr2.2066

**Published:** 2024-05-04

**Authors:** Baishali Tamuli, Sakshi Sharma, Meena Patkar, Subir Biswas

**Affiliations:** ^1^ Tumor Immunology and Immunotherapy, Advanced Centre for Treatment, Research and Education in Cancer (ACTREC) Tata Memorial Centre Kharghar, Navi Mumbai India; ^2^ Homi Bhabha National Institute Mumbai India

**Keywords:** gamma delta T cells, immunology, immunotherapy, MDSCs, tumor microenvironment, tumor‐associated macrophages

## Abstract

**Background:**

The tumor microenvironment of solid tumors governs the differentiation of otherwise non‐immunosuppressive macrophages and gamma delta (γδ) T cells into strong immunosuppressors while promoting suppressive abilities of known immunosuppressors such as myeloid‐derived suppressor cells (MDSCs) upon infiltration into the tumor beds.

**Recent findings:**

In epithelial malignancies, tumor‐associated macrophages (TAMs), precursor monocytic MDSCs (M‐MDSCs), and gamma delta (γδ) T cells often acquire strong immunosuppressive abilities that dampen spontaneous immune responses by tumor‐infiltrating T cells and B lymphocytes against cancer. Both M‐MDSCs and γδ T cells have been associated with worse prognosis for multiple epithelial cancers.

**Conclusion:**

Here we discuss recent discoveries on how tumor‐associated macrophages and precursor M‐MDSCs as well as tumor associated‐γδ T cells acquire immunosuppressive abilities in the tumor beds, promote cancer metastasis, and perspectives on how possible novel interventions could restore the effective adaptive immune responses in epithelial cancers.

## 1 INTRODUCTION

Epithelial cancer microenvironment is infiltrated with variable numbers of T cells,^1^, ^2^, ^3^, ^4^, ^5^ B lymphocytes,^1^, ^2^, ^3^, ^4^, ^5^, ^6^ Natural Killer (NK) cells,^4^, ^5^, ^7^ and different myeloid cells that include neutrophils, dendritic cells, monocytes, myeloid‐derived suppressor cells (MDSCs).^4^, ^5^, ^8^, ^9^, ^10^, ^11^ However, the antitumor functions of T cells or antibody production by tumor‐infiltrated B lymphocytes are often compromised due to the strong immunosuppressive environment with the tumor beds in the majority of human cancers. Hence, the immunosuppressive cell differentiation in the tumor microenvironment (TME) from tumor‐infiltrated myeloid precursors and γδ T cells has gained immense attention in the field. Myeloid cells which are primarily involved in the host defense mechanism^12^, ^13^ also contribute to the malignant progression of epithelial cancers when accumulated in the TME.^8^, ^14^ Among myeloid lineage cells, apart from terminally differentiated dendritic cells and monocytes that differentiate into macrophages in tissues under inflammatory conditions, MDSCs are the major contributor to an immunosuppressive microenvironment.^15^ Polymorphonuclear type of MDSCs commonly known as PMN‐MDSCs, resemble neutrophils in terms of their morphology and phenotypes,^9^ but they differ in their functions from neutrophils as classically activated neutrophils show antitumor functions while pathologically activated PMN‐MDSCs show an immunosuppressive nature in the TME.^16^ Precisely, both neutrophils and PMN‐MDSCs exhibit the phenotype CD14^−^CD11b^+^CD15^+^(CD66b^+^) cells in humans and CD11b^+^Ly6G^+^Ly6C^low^ cells in mice.^17^, ^18^ However, there have been reports of variations in the expression of some surface markers among neutrophils and PMN‐MDSCs. For example, compared to neutrophils, PMN‐MDSCs show higher expression of CD115 and CD244 in mice.^19^ It has recently been demonstrated that human PMN‐MDSCs highly express lectin‐type oxidized LDL receptor 1 (LOX‐1) which helps in distinguishing these cells from neutrophils in the peripheral blood and tumor tissues of patients in multiple cancer types.^20^ Reprogramming of neutrophils for the phenotypic switching to PMN‐MDSCs occurs due to G‐CSF, GM‐CSF, TGF‐β, TNF‐α, IFN‐γ, IFN‐β, and IL‐17 overexpressed by the tumor cells, cancer‐associated fibroblasts, and TAMs.^21^ M‐MDSCs are monocytic MDSCs and they are morphologically and phenotypically similar to monocytes.^9^ Monocytes show antitumor functions when they are differentiated to classical phagocytic macrophages (and perhaps dendritic cells) whereas M‐MDSCs show immunosuppressive functions and they can also differentiate into tumor‐associated macrophages (TAMs).^22^ Classically differentiated phagocytic macrophages are characterized by higher surface expression of class II MHC with low PD‐L1 expression while immunosuppressive macrophages differentiated from M‐MDSCs show higher PD‐L1 expression, very low MHC‐II and high Arginase 1 production.^23^, ^24^

PMN‐MDSCs use ROS for their immunosuppressive functions that require cell–to–cell contact with T cells which leads to antigen‐specific interaction between MDSCs and T cells,^25^ while M‐MDSCs produce NO, arginase, and immune suppressive cytokines among other mechanisms to suppress overall T cell responses without the requirement of antigen‐specific direct cell–to–cell contact.^26^

Protumoral macrophages comprise the most copious infiltrating leukocyte population in solid tumors.^10^ Blood monocytes differentiated from bone marrow (BM) and very similar monocytic MDSCs are the main sources of tumor‐associated macrophages (TAMs).^9^ Abnormal expansion and function of myeloid cells are characteristic of almost all solid tumors. Tumor‐derived inflammatory signals lead to the expansion of immature MDSCs with immunosuppressive activity from the bone marrow and accumulation in the periphery, and eventually, cells belonging to the myelomonocytic lineage (i.e., M‐MDSCs) settle in tumor beds or premetastatic niches.^11^, ^18^, ^27^ The TME has a direct effect on the plasticity of infiltrated myeloid cells.^10^, ^15^ M‐MDSCs rapidly transform into TAMs after migration to tumor beds,^27^, ^28^ and evidence from multiple sources suggests that M‐MDSCs^28^ and circulating monocytes^29^, ^30^ are determinants of macrophage accumulation and activities in epithelial solid tumor malignancies.

T lymphocytes are classified as alpha‐beta (αβ) and gamma‐delta (γδ) T cells depending on T cell receptors (TCRs) chemical composition.^31^ Unlike αβ T cells where the TCRs are composed of α and β polypeptides, TCRs of γδ T cells have γ and δ chains.^31^ Although γδ T cells include a very small proportion (around 0.5%–5.0%) of all T cells,^32^ they are capable of non‐MHC restricted antigen recognition and possess abundant cytokine secretion capacity.^32^ γδ T cells are involved in bridging the two arms of immunity, that is innate and adaptive immune responses.^33^ γδ T‐cells, exhibiting regulatory roles, infiltrate most solid tumors,^34^ and TME pose skewing conditions that transform them into immunosuppressive cells.^35^ While most immunotherapeutic strategies are aimed at utilizing effector functions of αβ T‐cells and antibody‐induced cytotoxicity, there is a strong rationale for rescuing the inhibitory activities of γδ T‐cells and/or TAMs against malignant progression. In this review, we discuss the molecular pathways as well as tumor environment‐induced polarization of immunosuppressive MDSCs, TAMs, and γδ T cells in human cancers.

## 2 TUMOR‐ASSOCIATED MACROPHAGES AND PRECURSOR MONOCYTIC MYELOID‐DERIVED SUPPRESSOR CELLS IN EPITHELIAL CANCER

MDSCs acquire their name based on their origin from myeloid cells and their immunosuppressive activity.^36^ Polymorphonuclear MDSCs (PMN‐MDSCs) and monocytic MDSCs (M‐MDSCs) are the two reasonably different groups of MDSCs^37^ that have been identified in both mice^38^ and humans.^39^ Phenotypic and morphologic similarities between PMN‐MDSCs and neutrophils exist, and M‐MDSCs are similar to monocytes and exhibit high plasticity.^37^ M‐MDSCs are more predominant in tumors and rapidly develop into tumor‐associated macrophages (TAMs).^9^, ^40^ Circulating blood monocytes are one of the major sources of TAMs, recruited into the tumor microenvironment.^41^ Cancer cells‐stimulated cancer‐associated fibroblasts (CAFs) secrete higher amounts of IL‐6 and GM‐CSF that induce circulating monocytes recruited into the tumor microenvironment to differentiate into immunosuppressive TAMs.^42^ Importantly, tissue‐resident macrophages have a major influence on tumor development as they are among the first cells to encounter transform cells at primary or metastatic sites.^43^ TAMs derived from tissue‐resident macrophages promote active proliferation and expansion of tumors in many cancers such as lung cancers and pancreatic ductal adenocarcinoma.^44^, ^45^

MDSCs were originally identified as suppressors of T cell activity in tumors^22^ and later demonstrated to govern tumor cell survival, invasion, angiogenesis, metastasis, and formation of premetastatic niches.^46^ Likewise, differentiated TAMs are often involved in cancer development.^47^ TAMs drive invasion of tumor cells, help maintaining the viability of cancer stem cells, and also promote angiogenesis within the primary tumor mass.^48^, ^49^ Importantly TAMs help in metastasis by supporting the extravasation of cancer cells from the primary tumors and help the tumor cells remain dormant at the site of metastasis.^50^ The immunosuppressive activity of TAMs is mainly associated with their capacity to inhibit cytotoxic T cells and natural killer cells' abilities to eradicate tumors.^51^ Sonic hedgehog (SHH), a ligand of Hedgehog (Hh) signaling by tumor cells is reported to drive immunosuppressive macrophage polarization from myeloid precursor cells and tumor development. Immunosuppressive macrophage polarization and their functions are mediated by Krüppel‐like factor 4 (Klf4) by inhibiting the synthesis of CXCL9 and CXCL10 by TAMs.^52^

The accumulation and differentiation of MDSCs are governed by two distinct but partially overlapping signals.^49^ The process primarily involves signals ignited by tumor‐derived factors, such as STAT3, Notch, NLRP3, IRF8, C/EBPβ, and so on, that are responsible for immature myeloid cell expansion without terminal differentiation.^49^, ^53^ Additional supportive signals are driven by STAT1, STAT6, NF‐κB pathway, PGE2, and COX2 expressed by tumor stroma.^53^ Multiple factors influence the recruitment and maintenance of a steady supply of MDSCs in a rapidly changing TME and are not specific to a cancer type.^9^, ^54^

**FIGURE 1:**
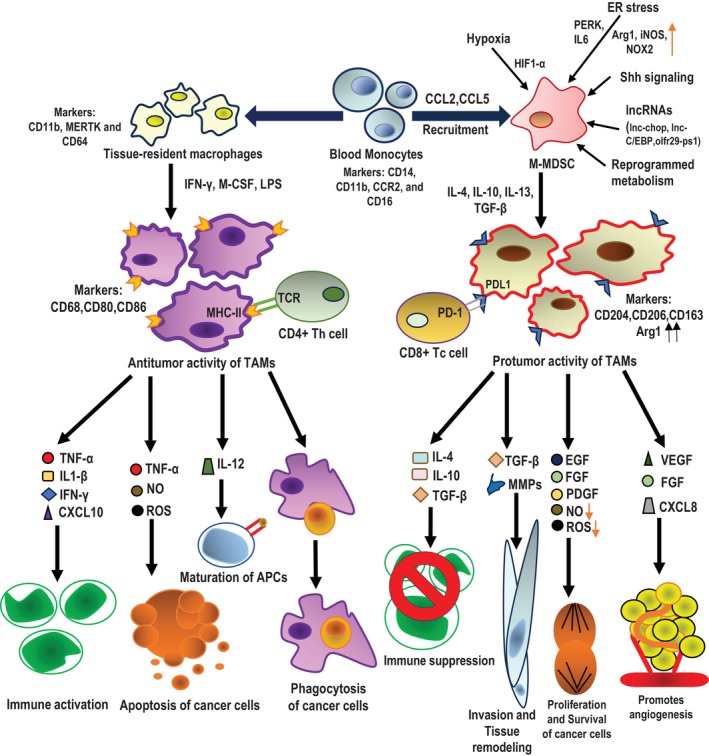
Recruitment of monocytes, M‐MDSCs into tumor microenvironment and differentiation of antitumor and protumor TAMs. Schematic representation showing how blood monocytes can migrate into malignant tumor tissues and form tissue‐resident macrophages that can play active antitumor activities by secreting different cytokines and chemokines that promote immune activation, maturation of APCs, and apoptosis or phagocytosis of cancer cells. Blood monocytes when recruited and differentiated as M‐MDSCs upon chemotactic infiltration by chemokines such as CCL2, CCL5, and so on, into the tumor microenvironment show potent immunosuppressive activity under the influence of hypoxia, ER stress, different lncRNAs, Shh signaling, and reprogrammed metabolism. After homing into the tumor beds, M‐MDSCs differentiate to TAMs with increased Arginase 1 expression and higher CD206, CD163, CD204 levels under the influence of IL‐10 and TGF‐β that secrete cytokines, chemokines, and other factors such as IL‐4, IL‐10, TGF‐β, MMPs, EGF, FGF, PDGF, VEGF, CXCL8, and so on, while show dampened expression of NO and ROS. M‐MDSC‐differentiated TAMs promote protumorigenic activities such as immune suppression, invasion and tissue remodeling, proliferation and survival of cancer cells, and angiogenesis. Monocytes‐derived tissue‐resident macrophages also differentiate into CD68^high^ CD80^+^ CD86^+^ MHC‐II^high^ TAMs, upon stimulation with IFN‐γ, M‐CSF, LPS, and so on, that primarily but not exclusively show antitumor functions such as immune activation, cancer cell apoptosis and phagocytosis and function as antigen‐presenting cells by secreting TNF‐α, IL1‐β, IFN‐γ, CXCL10, NO, ROS, IL‐12, and so on.

MDSCs are recruited to the tumor sites or the pre‐metastatic niches with the help of multiple chemokines (Figure 1) including CXCL1, CXCL2, CCL2, CXCL5, and CXCL12.^55^, ^56^ Additionally, CCL7, CXCL8, and CXCL12 have also been linked to monocyte recruitment.^9^, ^54^ Precisely, M‐MDSCs are found to be recruited by CCL2 and MIF^57^, ^58^, ^59^, ^60^, ^61^, ^62^, ^63^ while PMN‐MDSCs are recruited by CXCL1, CXCL2, and CXCL5.^64^, ^65^, ^66^, ^67^, ^68^ In colorectal cancer, SMAD4‐deficient colorectal cancer cells upregulate CCL15 secretion which favors the recruitment of MDSCs occur through CCL15/CCR1.^69^ Interestingly, SNAIL (Zinc finger protein SNAI1) helps in the recruitment of MDSCs by upregulating the expression of CXCL1/2 through the NF‐kB pathway.^70^ In hepatocellular carcinoma, hypoxia induces HIF expression which leads to the promotion of CCL26 transcription which eventually promotes recruitment of MDSCs through CX3CRI signaling.^71^ VEGF‐C, produced by human breast cancer cells, has been shown to interact with VEGFR3 on the surface of lymphatic endothelial cells, thereby promoting the production of several intracellular chemokines and increasing infiltrations of CXCR2^+^MDSCs.^72^ Importantly, FAS‐induced PGE2 aids in the recruitment of MDSC in lung cancer.^73^ Increased TNF‐α expression, transcription, and release from cancer cells due to PRKCI‐induced nuclear translocation of YAP1 attracts MDSCs into the tumor beds.^74^

## 3 CONTRIBUTORS THAT GOVERN MYELOID‐DERIVED SUPPRESSOR CELLS‐MEDIATED IMMUNOSUPPRESSION IN CANCER: HYPOXIA, ER STRESS, UPR

Hypoxia, specifically HIF1α, plays a crucial role in the differentiation and functionality of myeloid cells in the TME (Figure 1). For instance, HIF1α has been shown to promote differentiation of MDSCs toward immunosuppressive TAMs, and MDSCs lacking HIF1α instead acquire markers of dendritic cells.^28^ Hypoxia upregulates PD‐L1 expression on MDSCs driven by HIF1α. In the PD‐L1 proximal promoter, HIF1α directly binds with the hypoxia‐response element (HRE) and controls its expression.^75^ STAT3 that promotes differentiation of M‐MDSC to TAMs is sensitive to the activity of CD45 phosphatase in M‐MDSCs by hypoxia.^76^ In hepatocellular carcinoma, hypoxia causes overexpression of ENTPD2/CD39L1 that stabilizes HIF‐1 in the tumor cells which results in increased extracellular 5′ AMP.^77^, ^78^ Increased 5′ AMP drives MDSC‐maintenance while preventing their differentiation and stimulating synthesis of TGF‐β, arginase and release of nitric oxides.^77^, ^78^

MDSCs isolated from tumor‐bearing mice and cancer patients demonstrate that multiple endoplasmic reticulum (ER) stress indicators, including the transcription factors sXBP1 and C/EBP‐homologous protein (CHOP), are overexpressed and also show enlarged ER which is one of the hallmarks of ER stress.^36^ ER stress inducers increase ARG1, iNOS, and NOX2 expression, and therefore the immunosuppressive ability, in MDSCs (Figure 1). Correspondingly, ER stress reducers can reverse immunosuppression by MDSCs enhanced by ER stress.^79^ Mohammed E et al. recently demonstrated that the unfolded protein response (UPR) mediator, PKR‐like endoplasmic reticulum kinase (PERK) signaling is enhanced in tumor MDSCs^80^ (Figure 1). Through inhibiting STING signaling, PERK regulates MDSC‐driven immunosuppression in tumors.^80^ Deletion of PERK triggers SEC61β‐mediated immunogenic cell death (ICD) and drives CD8^+^ T cell‐mediated anti‐tumor responses.^80^, ^81^ CHOP, which is a known cellular stress sensor, promotes MDSCs‐induced T cell anergy by IL‐6 overexpression as well as STAT3 phosphorylation.^82^

## 4 ROLE OF LONG NON‐CODING RNAs ON MDSC DIFFERENTIATION

Several lncRNAs like lnc‐C/EBP homologous protein (*lnc‐chop*) and lnc‐C/EBP have been shown to play important roles in modulating MDSCs generation, recruitment, and immunosuppressive activities (Figure 1). *Lnc‐chop* present in MDSCs interacts with CHOP and C/EBPβ isoform, promotes C/EBP activation, and increases the expression of key molecules such as arginase‐1, NADPH oxidase 2, NO synthase 2, COX2, and so on, that cumulatively are associated with immunosuppressive functions of MDSCs in the TME. Importantly, *lnc‐chop* also enhances H3K4me3 enrichment in the promoter regions of these key molecules.^83^, ^84^ A lncRNA pseudogene Olfr29‐ps1, which is expressed in MDSCs, modifies the Olfr29‐ps1/miR‐214‐3p/MyD88 regulatory network that promotes differentiation and immunosuppressive functions of M‐MDSCs both in vitro and in vivo.^83^, ^85^

## 5 ROLE OF CELLULAR SIGNALING AND METABOLISM ON MDSC DIFFERENTIATION AND FUNCTION

Type I interferon (IFN1) receptor signaling has been shown to inhibit the suppressive activity of MDSCs.^86^ The decrease in the IFNAR1 chain of IFN1 receptor in MDSCs from tumor samples depends on p38 protein kinase activation that contributes to its immunosuppressive phenotype and has potent pro‐tumorigenic activity. Although, IFNAR1 loss alone is insufficient to convert neutrophils and monocytes to MDSCs but preventing its degradation inhibits the activities of MDSCs and has a significant anticancer effect.^86^ Interestingly, activated interferon signaling in astrocytes induces CCL2 that facilitates the recruitment of M‐MDSC into brain metastatic lesions.^87^ It is shown that SHP‐2 and PD‐1‐SHP‐2 signaling pathways limit the differentiation of myeloid cells by inhibiting phosphorylation of the transcription factors IRF8 and HOXA10 mediated by GM‐CSF, which stimulate monocyte/dendritic cell lineage commitment and myeloid differentiation, respectively.^88^ Different chemoattractants that activate Toll‐like/IL‐1 receptors, receptor tyrosine kinases, and G protein‐coupled receptors activate the tumor development, inflammation, and metastasis promoting PI3‐kinase isoform p110 in Gr1^+^CD11b^+^ myeloid cells.^89^ mTORC1 but not mTORC2 can regulate the differentiation of CD11b^+^ Ly6C^high^ M‐MDSCs and their immunosuppressive functions through the cellular metabolic pathway,^90^ while M‐MDSC differentiation is shown to be inhibited when glycolysis is blocked by 2‐deoxyglucose (2‐DG).^90^, ^91^ Accumulation of lipids in the TME leads to metabolic reprogramming of MDSCs from glycolysis to fatty acid oxidation^91^ (Figure 1). Oxidized lipids then serve as the main energy source for MDSCs, enhancing their immunosuppressive properties.^91^ Activation of tryptophan catabolite‐mediated aryl hydrocarbon receptor (AhR) causes massive mobilization of immunosuppressive MDSCs to tumor sites via stimulation of chemokines and their receptors like CXCR2.^92^

## 6 TUMOR MICROENVIRONMENT AND TUMOR‐ASSOCIATED MACROPHAGE CROSSTALK

After circulating monocytes and M‐MDSCs home to epithelial cancer beds they acquire immunosuppressive phenotypes and functional features. Cancer cells as well as other non‐malignant cells in the TME communicate bi‐directionally with M‐MDSCs and TAMs. Tumor‐promoting immunosuppressive TAMs have higher expression of certain markers and secretion of molecules associated with increased tumor growth which help cancer cells in escaping the immune surveillance^93^, ^94^, ^95^ (Figure 1). In lung cancer, CD204^+^ TAMs were shown to be associated with aggressive disease.^96^ In nasopharyngeal carcinoma, the number of FOXP3^+^ T regulatory cells (Treg) in the TME is positively correlated with immunosuppressive macrophages.^97^ Nasopharyngeal cancer cells induce immunosuppressive TAM polarization from precursor monocytes by secreting TGF‐β and IL‐10, which in turn produces chemoattractants that favor the accumulation of Treg.^97^ CAFs have also been proven to promote immunosuppressive macrophage polarization in multiple cancer types, for example, colorectal cancer,^98^ pancreatic ductal adenocarcinoma,^99^ hepatocellular carcinoma^100^ among others. In breast cancer, exosomes secreted by tumor‐associated mesenchymal stem cells induce differentiation of M‐MDSCs into more immunosuppressive TAMs with higher PD‐L1, CD206 expression, and arginase 1 activity.^101^ Interestingly γδ T cells are found to be necessary for immunosuppressive macrophage polarization during non‐cancerous experimental model of lung inflammation,^102^ while in cancer microenvironment MDSCs, mesenchymal cells, immunosuppressive TAMs and Tregs cumulatively prevent the infiltration and cytotoxic activity of γδT cells by inhibiting their secretion of IFN‐γ and promoting synthesis and release of immunosuppressive molecules.^103^, ^104^, ^105^

The unfolded protein response (UPR) mediator, PKR‐like endoplasmic reticulum kinase (PERK) is upregulated in macrophages in response to IL‐4 produced by Th2 and TME, promoting activation and proliferation of immunosuppressive TAMs. Activation of the PERK signaling cascade mediates phosphoserine aminotransferase (PSAT1) upregulation and increases serine biosynthesis through activation of stress‐responsive activating transcription factor 4 (ATF4). High serine biosynthesis balances α‐ketoglutarate (α‐KG) production required for JMJD3‐dependent histone demethylation that further promotes activation and proliferation of immunosuppressive TAMs.^106^

Oncostatin M (OSM), an inflammatory cytokine of the interleukin‐6 (IL‐6) superfamily, produced in hypoxic TME promotes the immunosuppressive phenotype of TAMs through mTOR signaling complex 2 (mTORC2)‐Akt1 axis.^107^ Hypoxia also triggers the secretion of CXCL8 in TAMs that activates the C‐X‐C Motif Chemokine Receptor 1/2 (CXCR1/2) on the plasma membrane of gastric cancer cells further activating the Janus kinase 1/Signal transducer and activator of transcription 1 (JAK/STAT1) signaling pathway. STAT1 transcription factor leads to the overexpression of IL‐10 that induces the immunosuppressive phenotype of TAMs and continues the overexpression of CXCL8 in a positive feedback loop aiding in the invasion and proliferation of gastric cancer cells.^108^

## 7 ANTITUMOR ACTIVITIES OF γδ T CELLS

**FIGURE 2:**
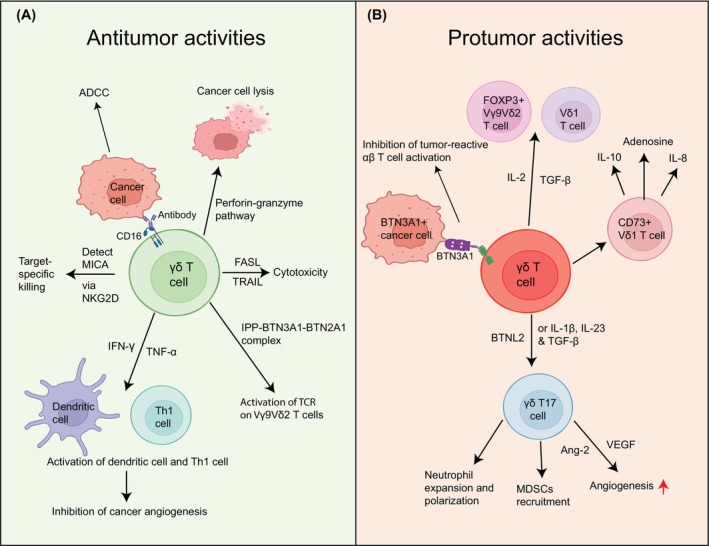
Antitumor and protumor activities of γδ T cells. Schematic representation of antitumor (A) and protumor (B) activities of γδ T cells. (A) γδ T cells mediate antitumor activity by binding with the Fc region of antibodies through the CD16 receptors on their surface and perform ADCC. They can do direct cancer cell lysis by perforin & granzyme secretion or cancer cell apoptosis by inducing the FASL‐TRAIL pathway. γδ T cells secrete IFNγ and TNFα that activate Th1, dendritic cells and inhibit angiogenesis. γδ T cells perform target‐specific killing by binding to MICA on cancer cells by NKG2D receptors on their surface. Isopentenyl pyrophosphate‐BTN3A1‐BTN2A1 complex, accumulated in cancer cells, can activate the TCR on Vγ9Vδ2 T cells. (B) γδ T cells help in cancer progression through polarization into immunosuppressive phenotypes such as FOXP3^+^ Vγ9δ2 T cells and Vδ1 T cells upon stimulated with TGF‐β and IL‐2 in the TME. Target specific killing and αβ T cells activating potential of Vγ9Vδ2 T cells could be inhibited by BTN3A1, without BTN2A1, expression on cancer cells. T17 γδ T cells recruit MDSCs into the tumor beds and also help in neutrophil expansion and polarization. They also contribute to angiogenesis by producing VEGF and Ang‐2. CD73^+^ Vδ1 T cells show immunosuppressive functions through their production of IL‐10, IL‐8, and adenosine. *Created with BioRender.com*.

γδ T cells lyse cancer cells via the perforin‐granzyme pathway,^33^ or can mediate direct cytotoxicity against tumor cells through Fas ligand (FASL) and TNF‐related apoptosis inducing ligand (TRAIL)^109^ (Figure 2A). Further, γδ T cells can also exert antitumor effects by binding to IgG antibodies through CD16 (FcγR III) Fc‐receptor present on their surface that promote antibody‐dependent cellular cytotoxicity (ADCC)^32^ (Figure 2A). Importantly, tumor infiltrated γδ T cells can detect stress‐induced molecules like MHC class I polypeptide‐related sequence A (MICA) via interaction with their natural killer group 2 member D (NKG2D) receptor and thereby can act as stress sensors,^109^ which further incites novel therapeutic implications for target‐specific killing^33^, ^110^ (Figure 2A). γδ T cells secrete cytokines IFN‐γ and TNF‐α that promote the activation of immune mediators such as Th1 cells and dendritic cells that inhibit angiogenesis in the tumor beds^32^, ^111^ (Figure 2A). Vγ9Vδ2 T cells can sense the intracellular accumulation of isopentenyl pyrophosphate (IPP) in the cancer cells due to the dysregulated mevalonate pathway.^109^ The intracellular domain of butyrophilin 3A1 (BTN3A1) molecule, which is expressed on the cancer cells binds to the accumulated IPP, and then interacts with BTN2A1 to bind and activate the TCR on Vγ9Vδ2 T cells^109^ (Figure 2A). In a recent study on head and neck cancer, it was suggested that BTN2A1 and BTN3A1 are the immediate ligands that promote the activation of γδ T cells.^112^

## 8 PROTUMOR ACTIVITIES OF γδ T CELLS

The cancer microenvironment modulates the γδ T cells into regulatory phenotypes as described above. This results in the generation of immunosuppressive γδ T cells that can impair the antitumor functions of multiple immune cells while enhancing the functions of immunosuppressive cells such as MDSCs.^32^ IL‐17 producing γδ T17 cells drive angiogenesis and tumor growth by inducing increased levels of VEGF and Ang‐2^109^ (Figure 2B). Further, it was demonstrated in a murine model of breast cancer that γδ T17 cells can recruit MDSCs of monocytic and granulocytic origin^113^ and induce neutrophil expansion and polarization leading to the development of pre‐metastatic immunosuppressive microenvironment^114^ (Figure 2B). Research shows that γδT17 cells can significantly contribute to the progression of human colorectal cancer by recruiting a larger number of MDSCs in the TME.^115^ A recent study provides evidence that γδ T cells activate pancreatic stellate cells (PSCs), resulting in the secretion of IL‐6, which might, in turn, promote pancreatic ductal adenocarcinoma (PDAC) development and progression.^116^ Breast cancer‐infiltrated CD73^+^ regulatory Vδ1 γδ T cell subpopulation exhibit their immunosuppressive functions by producing immunosuppressive molecules like IL‐8, IL‐10, and adenosine^117^ (Figure 2B).

## 9 EFFECTS OF TUMOR MICROENVIRONMENT ON TUMOR‐INFILTRATING γδ T CELLS

Similar to the tumor‐infiltrating myeloid population, TME also governs functions of tumor‐infiltrating γδ T cells turning them against anticancer immune responses, and promoteing cancer progression.^118^ A recent study has demonstrated that IL‐4, IL‐21, and TGF‐β in the TME favor the protumoral phenotype of γδ T cells.^119^ Additionally, γδ T cells can also be polarized into Th17‐like cells with protumor effects.^119^ Another recent study found IL‐17–producing γδ T cells in colorectal cancers, particularly the Vγ6Vδ1 clone showed extensive expansion in the tumor beds which resulted in tumor progression.^120^ Importantly, IL‐23, IL‐1β, and TGF‐β drive the polarization of Vγ9Vδ2 T cells to γδ T17 cells^121^ (Figure 2B). TGF‐β and IL‐2 together are involved in the development of FOXP3^+^ Vγ9Vδ2 and Vδ1 T cells,^119^ while IL‐21 drives their polarization toward a regulatory phenotype^119^ (Figure 2B).

Diving into the effects of TME on γδ T cells, there have been several recent studies on different cancer types. Antitumor cytotoxic functions and αβ T cells activating functions of Vγ9Vδ2 T cells are shown to be abrogated by BTN3A1 expression by the cancer epithelial cells in its spontaneous conformation without BTN2A1^122^ (Figure 2B). A recent study demonstrated that butyrophilin‐like protein 2 (BTNL2) in TME induces the production of IL‐17A by γδT17 cells, the most abundant population of γδ T cells,^115^ but not by the Vγ4 and Vγ6 γδ T cells^115^ (Figure 2B). Importantly, the production of IL‐17A by tumor‐infiltrating γδ T cells mediates infiltration of MDSC in the TME, and BTNL2 blockade reduces MDSC infiltration.^115^ Further, accumulation of kynurenine in indoleamine‐2,3‐dioxgenase (IDO) overexpressing tumor cells or stromal cells in the TME in PDAC impair degranulation and cytotoxic activities of γδ T cells.^123^ Interestingly, an amplification loop that involves γδ T cells‐IL‐17 and myeloid cells‐IL‐1β has been demonstrated to promote tumor growth.^124^ Mechanistically, neutralization of IL‐17, produced by γδ T cells, decreases infiltration of neutrophils and tumor burden with significant reduction of IL‐1β levels.^124^

## 10 REGULATION OF METASTASIS BY MDSCS, TAMS, AND γδ T CELLS

### 10.1 Myeloid‐derived suppressor cells

Myeloid‐derived suppressor cells (MDSCs) are one of the primary components responsible for an immunosuppressive tumor microenvironment. However, in addition to promoting immunosuppression, there has been growing evidence that MDSCs also play various non‐immunological roles, like promoting metastasis through the development of premetastatic niche, angiogenesis, and tumor cell invasion.^125^

Activation of the PI3K‐Akt–mTOR pathway by chemokine CCL3‐recruited MDSCs into the tumor microenvironment promotes breast cancer progression, migration, and invasion by the upregulation of MMP2 and MMP9.^126^ Higher infiltration of MDSCs induced by metastasizing breast cancer cells increases secretion of IL‐6 together with soluble IL‐6Rα.^127^ This exhibits a persistent upregulation of pSTAT3 in tumor cells and enhances the invasiveness of breast cancer cells.^127^ The tumor‐MDSC axis involving IL‐6 trans‐signaling can increase cancer cell aggressiveness in primary tumor locations and the metastasis to the lung by upregulating IL‐6 and sIL‐6Rα production.^127^

N6‐methyladenosine (m6A) in mRNA is shown to promote tumor growth and metastasis through the reprogramming of macrophages.^128^ Deletion of myeloid‐specific METTL3, which is the catalytic component of methyltransferase complex, reduces the translation of SPRED2 which ultimately increases the activation of STAT3 and NF‐kB via the ERK pathway promoting increased tumor growth and metastasis.^128^

Leukocyte immunoglobulin‐like receptor subfamily B member 4 (LILRB4, or gp49B the murine ortholog) which is expressed on many immune cells like activated T cells, monocytes, macrophages, dendritic cells, and NK cells, promotes polarization of MDSCs to a protumor phenotype and promotes cancer metastasis by secretion of pro‐tumor cytokines, increased immunosuppression of T cells, and angiogenesis.^129^ Further, MDSCs are one of the main sources of Wnt5A, a non‐canonical Wnt ligand that contributes to the accelerated metastatic spread of melanoma and decreases immunogenicity in the tumor microenvironment. Depletion of Wnt5A in the MDSCs inhibits infiltration of Tregs and impedes metastasis.^130^

A substantial increase in monocytic‐MDSCs recruited by chemokine CCL12 to the premetastatic lungs of tumor‐bearing mice has been observed. These recruited M‐MDSCs release IL‐1β even before the arrival of tumor cells which increases the expression of E‐selectin in the endothelial cells and tumor cell adherence.^131^

The interaction between MDSCs and tumor cells is mediated in part by cyclooxygenase‐2 (COX‐2) by activating the β‐catenin/TCF4 pathway, which suggests that inhibition of either COX‐2 or MDSCs may suppress metastasis of nasopharyngeal carcinoma.^132^ MDSCs were shown to enhance tumor lung metastasis by inducing the epithelial‐to‐mesenchymal transition (EMT) and promoting the invasion and migration of nasopharyngeal carcinoma cells.^132^ Interaction between MDSCs and nasopharyngeal carcinoma cells promotes TGFβ and nitric oxide (NO) production, which further upregulates COX‐2 expression subsequently activating MDSCs‐mediated EMT via the β‐catenin/TCF4 pathway.^132^

M‐MDSCs play a crucial role in reactivating dormant colorectal cancer cells to potential metastatic cells by secreting CCL2 that binds to the receptor CCR2 on these dormant cells and activates the JAK‐STAT3 signaling pathway that promotes the transition from dormant to highly metastatic cells.^133^

### 10.2 Tumor‐associated macrophages

TAMs produce numerous growth factors, cytokines, and chemokines that contribute to the immunosuppressive microenvironment. Apart from their immunosuppressive functions, TAMs also contribute to the invasion, extravasation, survival, intravasation, and colonization of tumor cells, all of which are critical processes in the metastasis of tumors.^50^ Importantly, TAMs secrete several pro‐angiogenic factors that include VEGF, TGF‐β, TNF‐α, IL‐8, IL‐1β, thymidine phosphorylase, platelet‐derived growth factor, and chemokines like CXCL8 and CCL2.^134^ Promotion of invasion and migration of breast cancer cells by TAMs to distant sites by secreting EGF which binds to EGFR on breast cancer cells has been observed. The breast cancer cells that migrate to the premetastatic niche interact with a different set of TAMs expressing distinct receptors from those identified at the primary site, which further promote tumorigenesis.^134^

TAMs promote EMT of colorectal cancer cells by secreting TGF‐β which leads to activating the Smad2,3‐4/Snail/E‐cadherin signaling pathway resulting in metastasis to the lung.^135^ TAMs have also been shown to promote invasion, migration, and metastasis in colorectal cancer via regulation of the JAK2/STAT3/miR‐506‐3p/FoxQ1 axis resulting in the production of CCL2 which in turn promotes the recruitment of more TAMs in a positive feedback loop.^136^ Lymph Node Metastasis Associated Transcript 1 (LNMAT1) is a long noncoding RNA associated with the prognosis and metastasis to the lymph nodes.^137^ LNMAT1 is upregulated in bladder cancer, which stimulates lymphangiogenesis and metastases by epigenetically activating CCL2 expression recruiting TAMs into the tumor beds, and facilitating lymphatic metastasis through the excretion of VEGF‐C^137^.

Interestingly, TAMs secrete CCL5 that promotes self‐renewal of prostate cancer stem cells (PCSCs) and enhances migration, invasion, and metastasis of prostate cancer cells by the activation of the β‐catenin/STAT3 signaling pathway.^138^ Silencing of CCL5 in TAMs inhibits bone metastasis and self‐renewal of PCSCs in vivo and therefore TAMs/CCL5 can be a novel target in predicting prognosis of prostate cancer and inhibiting metastasis.^138^

In the peritoneal cavity, TAMs may contribute to peritoneal dissemination in pancreatic cancer patients, where they interact with pancreatic cancer cells thereby increasing their migratory and invasive properties by inducing EMT which promotes metastasis and chemoresistance.^139^

TAMs‐secreted chitinase 3‐like protein 1 (CHI3L1), which is a glycoprotein highly expressed on cancer cells, promotes gastric and breast cancer metastasis by interacting and activating the interleukin‐13 receptor α2 chain (IL‐13Rα2) molecules present on cancer cells.^140^ Activated IL‐13Rα2 further activates the mitogen‐activated protein kinase signaling pathway, upregulating the expression of matrix metalloproteinases that promote tumor metastasis.^140^

### 10.3 γδ T cells

Reports on γδ T cells‐induced cancer metastasis are limited. IL‐17 producing γδ T cells (γδ T17 cells), which are induced by mammary tumors are involved in neutrophil polarization and expansion toward a CD8^+^ T cell‐suppressive phenotype, in turn, favor the establishment of metastases in distant organs.^141^ γδ T17 cells can also modulate adhesion molecules and increase endothelial cell permeability which in turn promotes tumor metastasis.^142^ Vδ1 T cells make up the majority of the tumor‐infiltrating γδ T cell population and release IL‐17 which aids in the spread of tumors by inducing the secretion of MMPs and VEGF by tumor cells. It has been observed that patients with advanced or metastasized malignancies have elevated levels of IL‐17.^143^, ^144^, ^145^ Mechanistically, IL‐17 ligation facilitates the in vivo progression of prostate cancer EMT, and hepatocellular carcinoma invasion through MMP‐2, MMP‐7, MMP‐9, and NF‐κB signal transduction.^146^, ^147^ When pancreatic cancer cells ligate to IL‐17, ERK signaling is directly up‐regulated which promotes cancer cell invasion and endothelial cell migration as well as the cancer cells' ability to survive in distant organs.^148^

## 11 NOVEL THERAPEUTIC APPROACHES WITH TUMOR‐ASSOCIATED MYELOID CELLS AND γδ T CELLS

**FIGURE 3:**
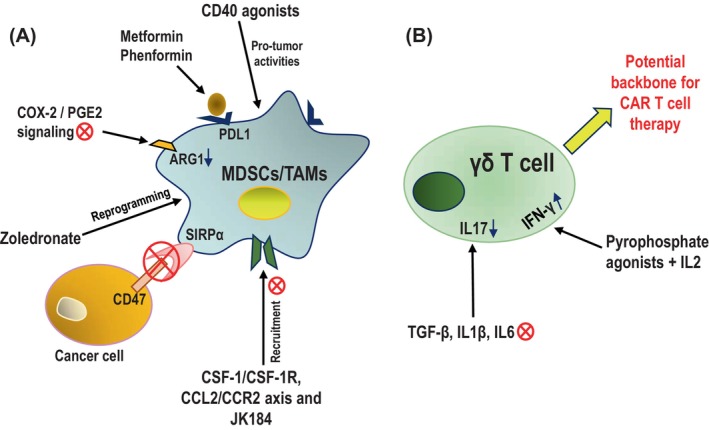
Therapeutic implications based on M‐MDSCs/TAMs and γδ T cells. (A) Schematic representation of approaches for targeting MDSCs and immunosuppressive TAMs. Metformin and Phenformin enhance the effectiveness of anti‐PD‐1 antibody‐mediated immune checkpoint blockade therapy. COX‐2/PGE2 signaling blockade represses effector factor ARG1 for MDSCs/TAMs‐mediated suppression. Blocking CD47 interaction with SIRPα rescues phagocytic abilities of TAMs whereas zoledronate can reprogram TAMs for reduced immunosuppressive abilities. CD40 agonists enhance the antitumor activities of TAMs. Recruitment of immunosuppressive MDSCs and TAMs in the TME could be abrogated by treatment with small molecules such as JK184 or by targeting CSF‐1/CSF‐1R and CCL2/CCR2 axis. (B) Schematic representation of approaches for rescuing cytotoxic functions of γδ T cells and their use as the backbone for novel CAR T development. Immunosuppressive γδ T cell polarization could be inhibited by targeting TGF‐β, IL‐1β, and IL‐6 with a decrease in IL‐17 production. Immunostimulatory IFNγ‐producing γδ T cell differentiation could be induced by pyrophosphate agonists with IL‐2. γδ T cells could be a better alternative compared to αβ T cells as a CAR T cell backbone preparation due to lesser toxicity where their innate cytotoxic abilities will be utilized.

Considering the immunosuppressive impacts of tumor‐associated MDSCs, macrophages, and γδ T cells, continuing efforts are being made to target these cells for rescuing protective immune responses at tumor beds. The effects of targeting MDSCs and immunosuppressive TAMs to reduce their number in the TME and impede their immunosuppressive functions have been performed independently by multiple groups.^149^, ^150^ Metformin and Phenformin, medications for controlling type 2 diabetes,^151^ inhibit the accumulation of immunosuppressive abilities in MDSCs and enhance the effectiveness of PD1 blockade therapy^152^, ^153^ (Figure 3A). T‐cell frequency and immune response can be improved by inhibiting COX‐2/prostaglandin E2 (PGE2) signaling, which suppresses MDSCs‐ and TAMs‐associated suppressive factors such as ARG1 expression or ROS production^154^ (Figure 3A). Three major categories of approaches have been explored for targeting TAMs: removing TAMs already present in the TME; inhibiting myelomonocytes recruitment; and reprogramming TAMs.^155^, ^156^, ^157^ However, the systemic removal of monocytes and macrophages by clodronate liposomes^101^ decouples overall immune function in the individual, and ensuring complete blockade of myelomonocytes‐infiltration into the tumor beds is challenging; therefore, currently, rescuing the phagocytic antitumor functions in TAMs is more in focus. Targeting CD47 on cancer cells,^158^ which binds with the SIRPα myeloid inhibitory receptor expressed on myeloid cells and makes them unresponsive against cancer cells,^159^, ^160^ is a novel way of rescuing phagocytic abilities of TAMs (Figure 3A). It is interesting to note that zoledronate, a bisphosphonate used to treat bone metastasis of cancer, targets TAMs, resulting in the depletion and reprogramming of TAMs (Figure 3A). Calcium zoledronate nanoparticles that are coated with lipids specifically target immunosuppressive TAMs and inhibit tumor growth.^161^ CD40 agonists monoclonal antibodies enhance secretion of proinflammatory cytokines by macrophage activation eliciting antitumor response of T cells.^162^ CD40 agonists promote anti‐tumor activities of macrophages in the tumor microenvironment that suppress proliferation and induce apoptosis in human pancreatic cancer cells^163^ (Figure 3A). Small molecule drug JK184 in combination with immune checkpoint blockades (ICBs) increases infiltration of T cells and secretion of inflammatory cytokines that inhibit recruitment of MDSCs and promotion of pro‐tumor activities of TAMs, reshaping the tumor immune microenvironment^164^ (Figure 3A). Recruitment of immunosuppressive TAMs in the TME could also be prevented by targeting CSF‐1/CSF‐1R axis^165^, ^166^, ^167^ and CCL2/CCR2 axis^168^, ^169^, ^170^, ^171^ (Figure 3A). A new approach to improve the phagocytic ability of TAMs has emerged due to the advancement of chimeric antigen receptor (CAR)‐based cell therapy,^172^ postulating that the CAR will secrete cytokines upon infusion that favor differentiation of antitumor proinflammatory TAMs.^173^, ^174^

Rescuing antitumor functions of tumor‐infiltrating γδ T cells and utilization of γδ T cells for cellular therapies against epithelial malignancies is a growing research area. It has been shown that the shift in the accumulation of intratumoral γδ T cells from antitumor IFNγ‐producing ones toward the protumor immunosuppressive IL‐17‐secreting γδ T cells increases through the stages of tumor progression.^175^ In theory, immunosuppressive γδ T cell polarization could be inhibited in situ by targeting cytokines that drive IL‐17 production, for instance, TGF‐β, IL‐1β, IL‐6, and so on.^176^ (Figure 3B). In vitro, treatment of Vγ9Vδ2 T cells with pyrophosphate agonists along with IL‐2 ensures differentiation of IFNγ^+^ IL‐17^−^ cells in the approaches of adoptive γδ T cells transfer^102^ (Figure 3B). To reduce the toxicity of CAR T cells in αβ T cells backbone, researchers have postulated to use γδ T cells as the backbone of CAR T cell preparation where the innate cytotoxic abilities will be utilized. Because of their dominant frequency in human blood,^177^ Vγ9Vδ2 T cells were the first choice,^178^ but later Vδ1 T cells were explored as a better CAR backbone.^179^ Most studies are still in infancy and in the coming years γδ CAR T cells may find an important place in anticancer therapeutics (Figure 3B).

## 12 CONCLUDING REMARKS

The effect of the TME is powerful and infiltrated γδ T cells and myelomonocytic cells or macrophages behave as directed by the TME. It is important to understand the immunosuppressive nature of the TME and tackle it in the best way possible. Immunooncology has been focused almost exclusively on αβ T cells with some recent understanding of the importance of infiltration of B lymphocytes^6^ and antibody production in epithelial cancers.^180^ However, there are multiple gray areas about the function and modes of action of tumor‐associated γδ T cells and macrophages. For rescuing activities of αβ T cells and antibody‐producing B cells in the tumor beds, it is important to rescue the antigen presentation functions of tumor‐associated myeloid cells and impede the suppressive functions of γδ T cells and myelomonocytic cells for achieving robust, effective adaptive immune response. Targeted approaches so far have been limited to the surface proteins of these cells. Novel small‐molecule inhibitors or antibody‐based therapies targeting unique intracellular or secreted molecules present in these suppressive cells, could synergize existing immunotherapies to achieve coordinated immune governance of solid tumor malignancies in humans.

## AUTHOR CONTRIBUTIONS

**Subir Biswas:** Conceptualization; funding acquisition; writing – original draft; methodology; writing – review and editing; visualization; project administration; supervision; investigation. **Baishali Tamuli:** Writing – original draft; funding acquisition; writing – review and editing; methodology. **Sakshi Sharma:** Writing – original draft; writing – review and editing; methodology. **Meena Patkar:** Writing – review and editing.

## CONFLICT OF INTEREST STATEMENT

The authors do not have any competing interests.

## ETHICS STATEMENT

Not applicable.

## Data Availability

Data sharing is not applicable to this article as no new data were created or analyzed in this study.
